# Supplementary figures and images for: The HIV-1 capsid serves as a nanoscale reaction vessel for reverse transcription
Source: PLoS Pathog. 2024 Sep 3;20(9):e1011810. doi: 10.1371/journal.ppat.1011810 (PMC11398657; doi:10.1371/journal.ppat.1011810)

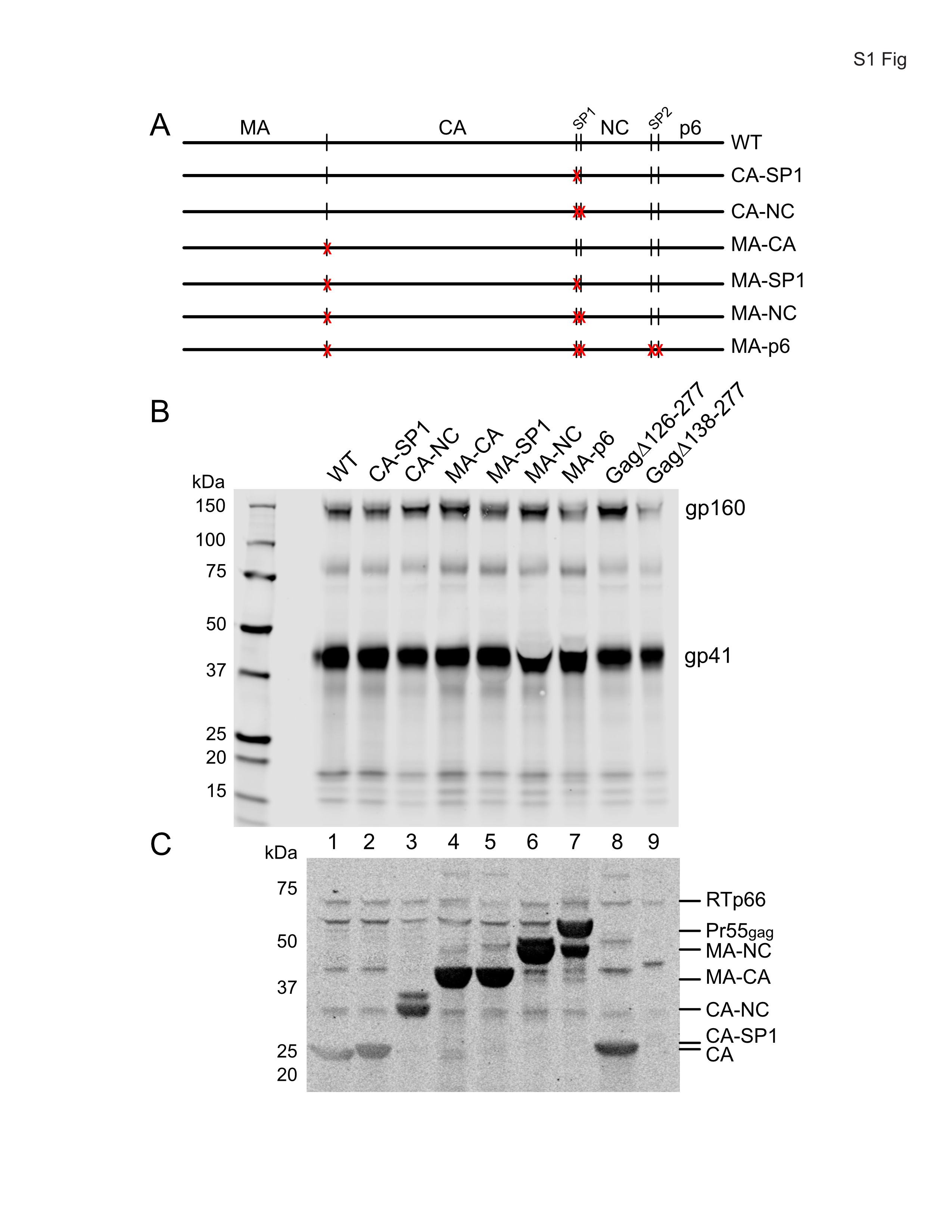

Supplement: S1 Fig — (A) Depiction of HIV-1 mutants containing mutations preventing cleavage at various sites in Gag. All mutants shown are incapable of forming a mature, stable, conical capsid. (B and C) Immunoblot of pelleted mutant virions with blocked cleavage sites and two mutants with large deletions in CA. Panel B shows a scan of the blot following detection of gp41, and C shows the blot following reprobing with polyclonal human antiserum to HIV-1 (HIV-Ig). (TIFF) [file ppat.1011810.s001.tiff]
